# The Latent Stem Cell Population Is Retained in the Hippocampus of Transgenic Huntington's Disease Mice but Not Wild-Type Mice

**DOI:** 10.1371/journal.pone.0018153

**Published:** 2011-03-24

**Authors:** Tara L. Walker, Geoff W. Turnbull, Eirinn W. Mackay, Anthony J. Hannan, Perry F. Bartlett

**Affiliations:** 1 Queensland Brain Institute, The University of Queensland, Brisbane, Australia; 2 Howard Florey Institute, University of Melbourne, Melbourne, Australia; University of Nebraska Medical Center, United States of America

## Abstract

The demonstration of the brain's ability to initiate repair in response to disease or injury has sparked considerable interest in therapeutic strategies to stimulate adult neurogenesis. In this study we examined the effect of a progressive neurodegenerative condition on neural precursor activity in the subventricular zone (SVZ) and hippocampus of the R6/1 transgenic mouse model of Huntington's disease (HD). Our results revealed an age-related decline in SVZ precursor numbers in both wild-type (WT) and HD mice. Interestingly, hippocampal precursor numbers declined with age in WT mice, although we observed maintenance in hippocampal precursor number in the HD animals in response to advancement of the disease. This maintenance was consistent with activation of a recently identified latent hippocampal precursor population. We found that the small latent stem cell population was also maintained in the HD hippocampus at 33 weeks, whereas it was not present in the WT. Our findings demonstrate that, despite a loss of neurogenesis in the HD hippocampus *in vivo*, there is a unique maintenance of the precursor and stem cells, which may potentially be activated to ameliorate disease symptoms.

## Introduction

It is now well established that the hippocampus and subventricular zone (SVZ), even in humans, continue to undergo neurogenesis throughout life, producing neural precursors that differentiate into mature neurons. A number of recent studies have also demonstrated the brain's ability to respond to damage such as trauma and disease through an up-regulation in neurogenesis [1], [2]. In Huntington's disease (HD), a neurodegenerative condition characterized by motor, psychiatric and cognitive deficits, proliferation in neurogenic regions and the production of neurons have also been reported [3], [4]. Transgenic R6 lines of HD mice have been well characterized and model many HD symptoms and neuropathology, including progressive cognitive decline, motor deficits, weight loss and neuronal dysfunction [5]–[10]. It has also been demonstrated that environmental enrichment and antidepressant treatment delay both the onset and progression of disease in the R6/1 transgenic mouse model of HD [6], [8], [11], with both experimental manipulations up-regulating hippocampal neurogenesis and promoting maturation of migrating neurons [11], [12].

In the present study, we examined the *in vitro* hippocampal and SVZ precursor activity of age-matched HD and wild-type (WT) mice, at five time points over a 33 week period, to determine what changes occurred in response to disease progression. We found that there was an increase in hippocampal, but not SVZ, precursor numbers as the disease advanced. This up-regulation was consistent with the activation of a normally latent hippocampal precursor population [13], with no further activation being observed in the HD hippocampus following *in vitro* depolarization. Most importantly, we found that a small subset of the precursors which have stem cell activity were maintained in the HD hippocampus but were not found in the hippocampus of WT littermates.

## Materials and Methods

### Mice and behavioral tests

Mice were derived from breeding of R6/1 hemizygous male mice, obtained from a colony at the Howard Florey Institute (Melbourne, Australia), with WT strain-matched B6CBAF1/J females. The University of Queensland Animal Ethics Committee approved all procedures (approval numbers SBMS/QBI/289/05/UQ and QBI/453/05/BREED). Animals were examined for signs of HD, with behavioral data being collected from cohorts of HD and WT mice at 16, 22 and 30 weeks of age. Animals were weighed and then examined for rear-paw clasping (RPC), an indication of a HD phenotype in the R6/1 model, by suspending them by their tail briefly [6], [14]. Motor performance was then assessed using an accelerating rota-rod (UGO Basile model 7650; Sandown Scientific, Hampton, UK). Animals were scored for time spent on the rota-rod as it accelerated from 4 rpm to 40 rpm over a 3 minute period. Mice were then sacrificed by cervical dislocation and the whole brain removed and weighed.

### Neurosphere assays

Primary neurospheres were generated as described previously [15], [16]. Briefly, the tissue was enzymatically digested with 0.1% trypsin-EDTA (Gibco/Invitrogen, Eugene, OR) for 7 minutes at 37°C, followed by addition of 0.014% w/v trypsin inhibitor (type I-S from soybean; Sigma-Aldrich, Sydney, Australia) dissolved in Hepes-buffered minimum essential medium (HEM). The digested tissue was centrifuged at 100 rcf for 5 minutes, after which the pellet was resuspended in 1 ml of neurosphere growth medium, mechanically triturated, then filtered through a 40 µm cell sieve (Falcon/BD Biosciences, Sydney, Australia). The neurosphere growth medium consisted of mouse NeuroCult™ NSC Basal Medium plus mouse NeuroCult™ NSC Proliferation Supplements (StemCell Technologies, Vancouver, Canada) with 0.2% bovine serum albumin (Gibco/Invitrogen) and 2 µg/ml heparin (Sigma-Aldrich). The following growth factors were also included: 20 ng/ml purified mouse receptor-grade epidermal growth factor (BD Biosciences, Sydney, Australia) and 10 ng/ml recombinant bovine basic fibroblast growth factor (Roche, Basel, Switzerland). Cells were plated at a density of one hippocampus (approximately 2000 cells per well) or SVZ (approximately 1000 cells per well) per 96-well plate (Falcon/BD Biosciences) with 200 µl of neurosphere growth medium per well. For the depolarization experiments, additional KCl was added at the time of plating the primary cells to give a final concentration of 15 mM. Previous experiments have demonstrated the neurosphere-forming activity in the hippocampus to be approximately one neurosphere for every 9227 cells plated and in the SVZ to be one neurosphere for every 766 cells plated [16]. Therefore, at these densities it is expected that any neurosphere formed will be clonally derived. Primary hippocampal cells were incubated for 10 days and SVZ cells for 7 days in humidified 5% CO_2_. Primary neurospheres (≥50 µm in diameter) were then counted and sized using a standard light microscope with an eyepiece graticule. Results of the neurosphere counts were expressed as mean ± standard error, and statistical analysis was performed using a Students *t*-test (two sample assuming equal variance).

### Neurosphere differentiation and immunocytochemistry

Neurospheres were plated onto poly-d-lysine coated coverslips in NeuroCult™ NSC basal medium containing mouse NeuroCult™ NSC proliferation supplements and 2% fetal calf serum (Sigma-Aldrich) without growth factors. They were then allowed to differentiate at 37°C for 5 days in humidified 5% CO_2_ until flattened and adherent. Differentiated neurospheres were fixed with 4% paraformaldehyde (PFA; Sigma-Aldrich) in 0.1 M phosphate buffered saline (PBS) at room temperature for 30 minutes. After washing with PBS, neurospheres were incubated for 60 minutes at room temperature with blocking solution: 5% fetal calf serum plus 5% normal goat serum (Sigma-Aldrich) in 0.1 M PBS containing 0.1% Triton X-100 (Sigma-Aldrich). The blocking solution was replaced with fresh solution containing mouse monoclonal βIII tubulin antibody (1∶2000; Promega, Madison, WI) plus rabbit polyclonal glial fibrillary acidic protein (GFAP) antibody (1∶500; DakoCytomation, Carpinteria, CA) and incubated for 60 minutes at room temperature. Cells were washed with PBS and incubated for 30 minutes at room temperature in blocking solution containing Alexa Fluor 568 anti-mouse antibody (1∶1000; Molecular Probes/Invitrogen), Alexa Fluor 488 anti-rabbit antibody (1∶1000; Molecular Probes/Invitrogen) and 4,6-diamidino-2-phenylindole (DAPI; 1∶5000; Sigma-Aldrich). Following washing with PBS, slides were coverslipped with fluorescence mounting medium (DakoCytomation) before viewing on a Zeiss upright fluorescence microscope. Images were captured by a digital camera linked to a computer running Axioscope version 4 (Zeiss, Gôttingen, Germany).

### Quantification of *in vivo* neurogenesis

To quantify bromodeoxyuridine (BrdU)- and BrdU/GFAP-positive cells, mice received a single intraperitoneal (i.p.) injection of BrdU (45 mg/kg body weight), dissolved in 0.07 N NaOH in 0.9% NaCl (Sigma) 2 hours prior to perfusion. For quantification of BrdU/doublecortin (DCX)-positive cells, mice received 6 i.p. injections of BrdU (45 mg/kg body weight), once every 2 hours, 10 days prior to perfusion. Animals were perfused with 0.1 M PBS followed by 4% PFA. Brains were removed and incubated overnight in 4% PFA, followed by further overnight incubations in 20% sucrose then 30% sucrose in 0.1 M PBS at 4°C. Frozen sections (50 µm) were cut using a sliding microtome. Every sixth section (approximately 7 sections per animal) was stained and mounted. For BrdU immunohistochemistry, sections were first denatured with 2N HCl for 30 minutes at 37°C, then washed briefly in PBS. All sections were incubated for 60 minutes at room temperature with blocking solution as described above. Sections were then incubated, with rat anti-BrdU antibody (1∶100; Auspep, Melbourne, Australia), rabbit polyclonal DCX antibody (1∶500; Abcam, Cambridge, MA) or rabbit polyclonal GFAP antibody (1∶500; DakoCytomation), at 4°C overnight. Following washes with PBS, sections were incubated for 40 minutes at room temperature in blocking solution containing either Alexa Flour 488 donkey anti-rat secondary antibody (1∶1000), or Alexa 568 anti-rabbit antibody (1∶1000; Molecular Probes/Invitrogen), and DAPI (1∶1000). After washing with PBS, the slides were coverslipped with fluorescence mounting medium and all BrdU-, DCX-, BrdU/GFAP- or BrdU/DCX-positive cells in the dentate gyrus of the hippocampus were counted using a Zeiss AxioObserver with Colibri illumination.

## Results

**Figure 1:**
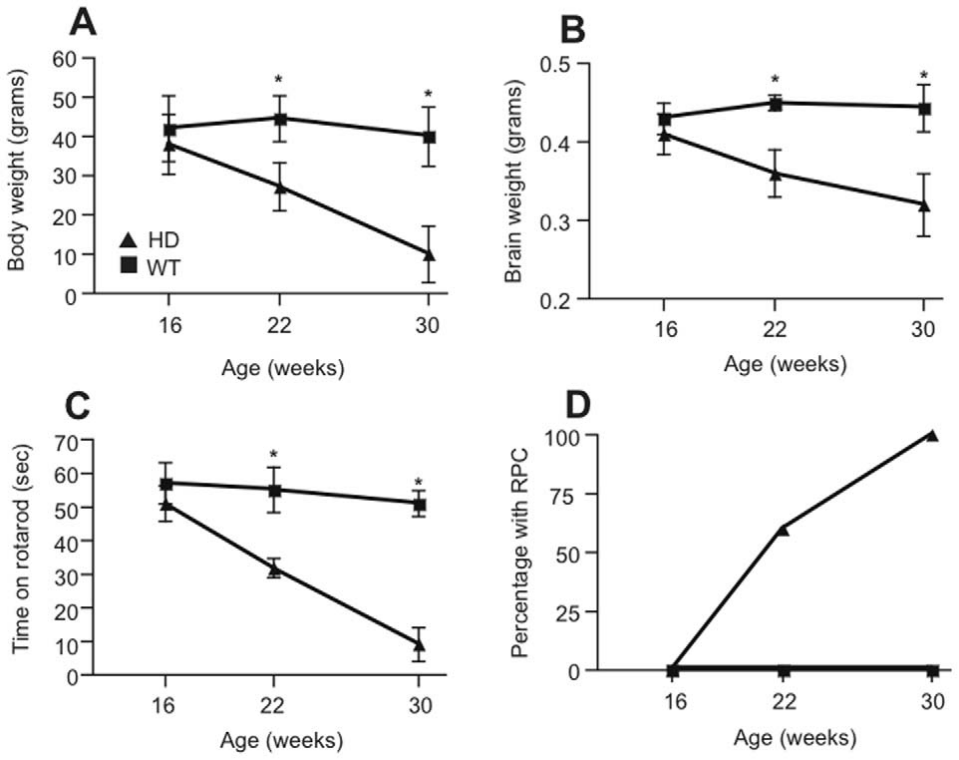
Correlation between behavioral deficits in HD and WT mice and disease progression. (A) Body weights of HD mice are significantly lower than those of their WT littermates at 22 and 30 weeks. (B) HD mice had lower whole brain weights at 22 and 30 weeks than their WT littermates. (C) At 22 and 30 weeks HD mice have poorer motor ability and fall off the accelerating rota-rod at lower speeds than WT littermates. (D) The percentage of HD mice expressing the rear-paw clasping (RPC) motor phenotype increases to 100% by 30 weeks. Data are expressed as mean ± s.e.m, *n*≥3 animals, *p≤0.05.

We first examined the *in vivo* effects of HD progression in the R6/1 strain by measuring four parameters: body weight, brain weight, percentage of mice with RPC and time spent on a rota-rod. The results showed that at 16 weeks of age HD mice were indistinguishable from their WT littermates (Fig. 1). From 22 weeks, however, HD mice showed a significant drop in body weight (Fig. 1A), brain weight (Fig. 1B), time spent on the rota-rod apparatus (Fig. 1C), and 60% of the HD group displayed the RPC phenotype (Fig. 1D). At 30 weeks of age the HD mice were even more severely affected, and 100% of the group exhibited the RPC motor phenotype. In contrast, there were no significant differences observed in any of the parameters tested in WT animals across the three ages.

**Figure 2:**
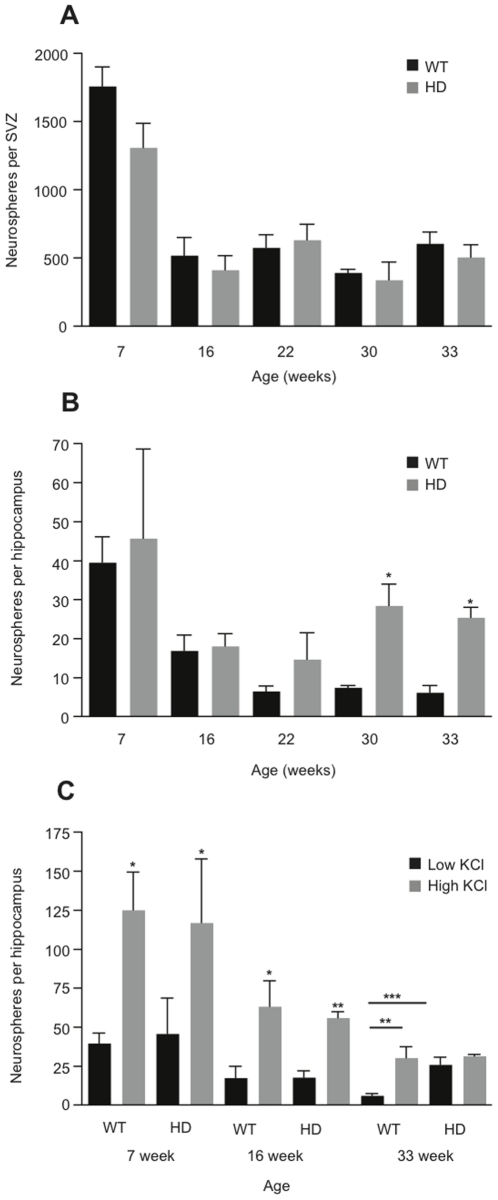
Increased precursor activity in the hippocampus but not the SVZ of symptomatic HD mice. (A) Similar numbers of SVZ-derived neurospheres were generated from HD and WT littermates at all time points examined. (B) A significant increase in the number of hippocampal-derived neurospheres in HD mice compared to WT littermates was observed at 30 (*n* = 5) and 33 weeks of age (*n* = 4). (C) Younger (16-week old) HD mice generated a similar number of hippocampal neurospheres to their WT littermates and the number of neurospheres could be increased by the addition of depolarizing levels of KCl (*n* = 3). More neurospheres were generated from older symptomatic HD mice (33 weeks; *n* = 4) than the corresponding WT mice. However, unlike the WT mice, which show a significant increase in neurosphere number following *in vitro* depolarization, no further increase was observed in the HD mice in the presence of additional KCl. All data are expressed as mean ± s.e.m, *p≤0.05, **p≤0.01, ***p≤0.001.

We examined precursor activity across advancing age and disease by performing *in vitro* neurosphere assays with tissue harvested from the hippocampus and SVZ of HD and WT littermates and performing *in vitro* neurosphere assays (Bull and Bartlett, 2005). Results showed a drop in neurosphere number from the SVZ of WT and HD mice over time (Fig. 2A). The number of SVZ-derived neurospheres from WT and HD mice decreased from 1762±140 to 607±84 and 1311±178 to 507±92, respectively, as the mice aged from 7 to 33 weeks (Fig. 2A). However, there was no significant difference in the total number of SVZ-derived neurospheres between HD and WT genotypes at any time point examined.

Similar to the finding in the SVZ, there was no difference in hippocampal neurosphere numbers between HD and WT littermates at stages prior to the onset of motor deficits (7 or 16 weeks) (Fig. 2B). Surprisingly, as the mice aged and became symptomatic there was a significant difference in hippocampal precursor number in the HD mice compared to the aged-matched WT controls (Fig. 2B). This increase in hippocampal neurosphere activity was first observed at 22 weeks, with HD mice showing an approximately 2-fold increase in neurosphere numbers compared to their WT littermates. With disease progression the up-regulation of *in vitro* precursor activity increased still further, with mice aged 30 and 33 weeks recording a significant 3.5- and 4-fold increase in neurosphere number respectively, compared to their WT littermates (Fig. 2B).

Recently, we demonstrated that the adult hippocampus contains a large number of latent precursors, including a self-renewing stem cell population, which only becomes activated following depolarization [13]. We therefore next explored whether it was this precursor cell population that was being activated in the HD mice. At 7 and 16 weeks, prior to the emergence of symptoms, both WT and HD mice had a similar number of neurosphere-forming cells (Fig. 2C). In addition, *in vitro* depolarization increased the number of hippocampal neurospheres generated from both genotypes approximately 3-fold (Fig. 2C). In contrast, at 33 weeks there was a significant increase in the number of neurospheres generated, under low K^+^ culture conditions, from the hippocampus of HD mice compared to WT littermate controls (Fig. 2C). Consistent with our previous findings in an *in vivo* seizure model [13], the precursor population in the 33-week old HD hippocampus was also completely activated, as no significant further increase in neurosphere number occurred following KCl depolarization *in vitro* (Fig. 2C). In addition, the number of neurospheres cultured in control conditions (low K^+^) from the 33-week old HD mice was similar to that obtained in depolarizing conditions (high K^+^) from WT littermates, further indicating complete activation of the precursor population in the HD hippocampus (Fig. 2C).

**Figure 3:**
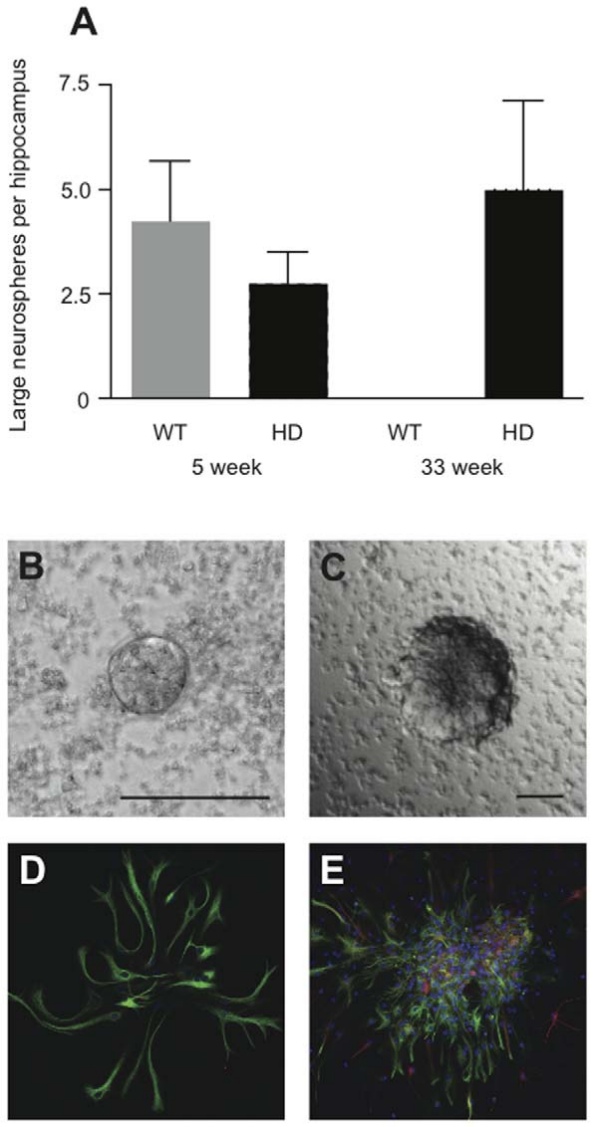
Large stem cell-derived neurospheres were generated from 33-week old HD hippocampus, but not WT hippocampus. (A) Similar numbers of large (≥250 µm in diameter) hippocampal neurospheres were generated from HD (*n* = 4) and WT (*n* = 4) mice at 5 weeks of age. However, by 33 weeks, large neurospheres could only be generated from the HD hippocampus (*n* = 4) and not the WT (*n* = 5) hippocampus. Data are expressed as mean ± s.e.m. Representative images of a small progenitor-derived neurosphere from a 33-week old WT hippocampus (B) and a large stem cell-derived neurosphere from a 33-week old HD hippocampus (C). Small neurospheres generated from the 33-week old WT hippocampus gave rise exclusively to astrocytes (shown in green; D), whereas the large neurospheres generated from the 33-week old HD hippocampus gave rise to astrocytes (green) and a small number of neurons (red; E). Scale bars in B and C are 100 µm.

It is known that neurosphere size is directly related to proliferative capacity and we have previously shown that only very large hippocampal neurospheres, with diameters over 250 µm, are derived from stem cells [13]. At pre-symptomatic time-points, in addition to activating a progenitor cell population, KCl depolarization was also able to activate the previously characterized latent stem cell population, as evidenced by the formation of a number of large (>250 µm diameter) neurospheres from both WT and HD hippocampi (Fig. 3A). We have previously reported that in the hippocampus of older WT mice we were no longer able to activate this stem cell population but rather could activate only a more restricted progenitor population [13]. In accordance with this observation, no large stem cell-derived neurospheres could be generated from 33-week old WT mice in the present study. Surprisingly, large neurospheres were generated in the 33-week old HD cultures following depolarization *in vitro* (Fig. 3A), indicating that the latent stem cell population was retained in the HD hippocampus and could be activated. As expected, when the large stem cell-derived neurospheres generated from pre-symptomatic 7- and 16-week old HD and WT mice were differentiated and stained they gave rise to GFAP-positive astrocytes as well as a small number of βIII-tubulin-positive neurons. Although the smaller progenitor-derived neurospheres generated from the 33-week old WT animals only produced astrocytes (Fig. 3B, D), the large neurospheres generated from the 33-week old HD hippocampus gave rise to a small number of neurons (Fig. 3C, E).

**Figure 4:**
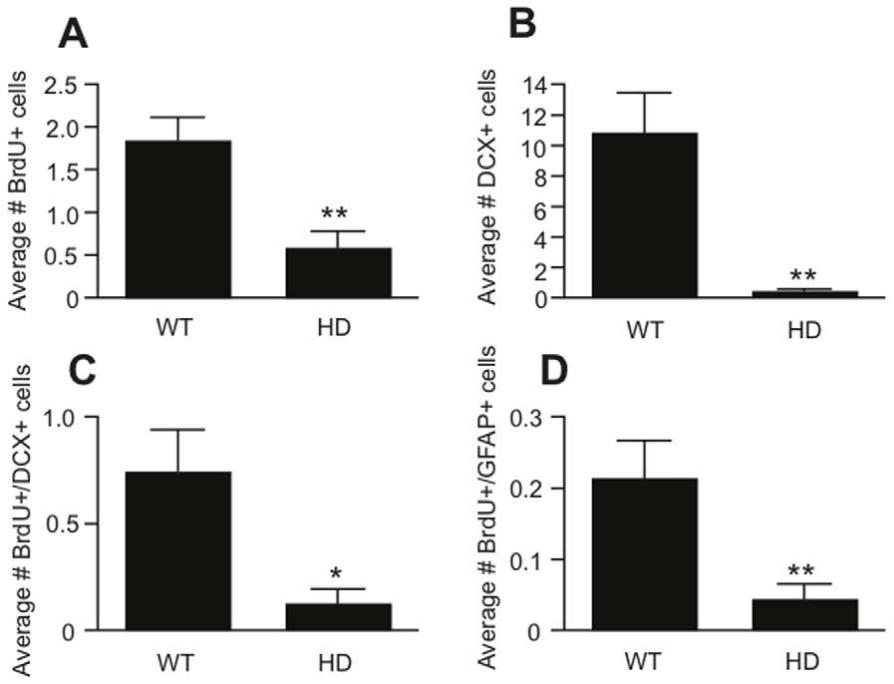
Quantification of *in vivo* neurogenesis in the hippocampus of symptomatic HD and WT mice. Significantly fewer BrdU-positive (A, *n* = 8 HD and *n* = 9 WT animals), DCX-positive (B, *n* = 5 HD and *n* = 6 WT animals), BrdU/DCX double-positive (C, *n* = 5 HD and *n* = 6 WT animals) and BrdU/GFAP double-positive (D, *n* = 8 HD and *n* = 9 WT animals) cells were observed in the hippocampus of 33-week old HD mice compared to WT littermates. Data are expressed as mean ± s.e.m, *p≤0.05, **p≤0.01.

**Figure 5:**
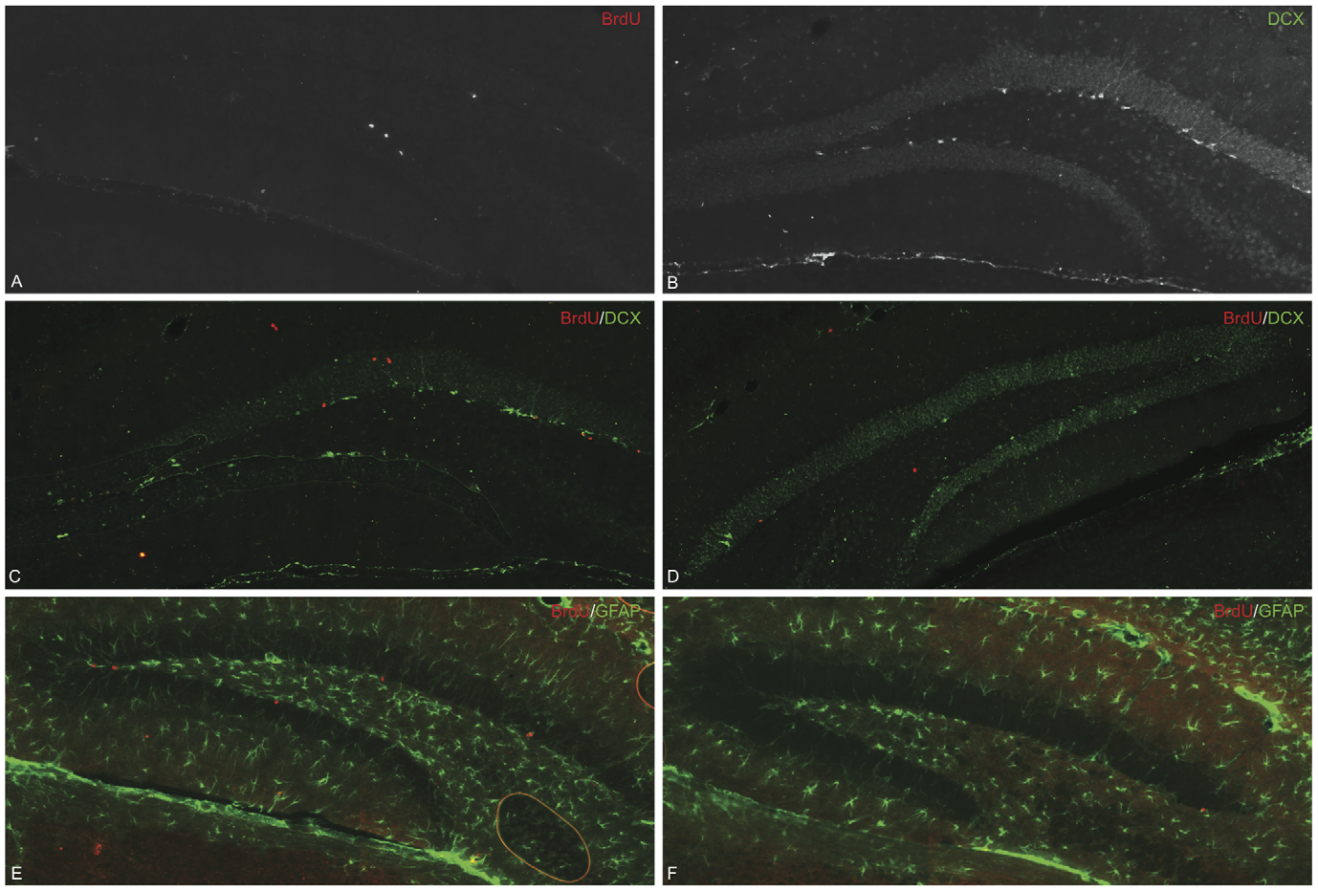
Representative images from the immunstaining of 33-week-old WT and HD hippocampus. Representative images of BrdU (A) and DCX (B) staining in the WT hippocampus. Composite images of BrdU (red) and DCX (green) double-staining in the WT (C) and HD (D) hippocampus. Composite images of BrdU (red) and GFAP (green) double-staining in the WT (E) and HD (F) hippocampus. All images were taken using the 20x objective.

Finally, we examined whether the increase in precursor activity that we observed in the 33-week old HD mice using the *in vitro* neurosphere assay resulted in an increase in hippocampal neurogenesis *in vivo*. To address this we stained hippocampal sections taken from 33-week old HD and WT animals, which had received BrdU injections, with markers for proliferating cells (BrdU), newly born neurons (DCX), and astrocytes/stem cells (GFAP). Interestingly, we found a significant decrease in BrdU-, DCX-, BrdU/DCX- and BrdU/GFAP-positive cells in the symptomatic HD hippocampus compared to the WT hippocampus (Fig. 4, Fig. 5).

## Discussion

Here we have shown that as HD mice became symptomatic there is a significant increase in *in vitro* hippocampal precursor activity compared to that of aged-matched WT controls. Most importantly, we have also found that although a latent stem cell population is no longer present in the WT hippocampus after 30 weeks of age, it is maintained in the HD hippocampus. In contrast, precursor numbers in the SVZ decrease with age, although no effect of HD disease progression was observed.

Our observation that HD progression had no effect on SVZ precursor numbers was in contrast to a recent report by Batista and colleagues [17], who showed a progressive increase in SVZ activation in R6/2 HD mice with disease progression. The lack of SVZ activation in our study could be due to the fact that the Batista study was examining R6/2 animals [5], which represent a more severe model, bearing a closer resemblance to juvenile-onset HD. Another study by Curtis et al. [3] also reported an increase in SVZ neurogenesis; however, these authors examined post-mortem tissue from patients who had been symptomatic for many years, such that a large number of striatal neurons would have already died. Therefore, the observed increase may have been due to a late and failed response to massive cell death. At the time-points used in the present study there would be very little or no cell death in the R6/1 striatum or other areas, explaining why we see no increase in the number of SVZ precursors. This suggests that the progenitors are reactive to their environment and are capable of initiating varying degrees of activation depending on the level of damage.

We have recently demonstrated that depolarization resulted in the activation of a latent precursor population in the adult mouse hippocampus [13], and that depolarizing levels of KCl led to the activation of a small subpopulation of stem cells with the capacity to generate very large neurospheres [13]. More importantly, we have also shown that this latent hippocampal progenitor population can be activated *in vivo* in response to prolonged neural activity, such as that found in status epilepticus. Although hippocampal neurogenesis has been shown to decrease with age [18], [19], we have demonstrated here that in the oldest symptomatic HD animals tested (33 weeks) there was in fact a significant increase in precursor numbers *in vitro* to levels greater than those recorded in much younger WT animals (16 weeks). While the latent stem cell population (as evidenced by the formation of neurospheres >250 µm in diameter) was lost in the WT animals at 33-weeks of age, it was maintained in HD animals of the same age.

It has previously been shown that symptomatic HD mice are prone to epileptic seizures, and there is also evidence that the increased electrical activity that occurs during seizures can lead to increases in neurogenesis in the dentate gyrus. This could potentially be one mechanism for the increased precursor activity we observe in the hippocampus following HD disease progression. It is important to note, however, that similar to the results we previously observed following status epilepticus, although we could culture more neurospheres from the symptomatic HD hippocampus we were unable to culture any of the large neurospheres prior to depolarization *in vitro*. However, following depolarization, activation of the latent stem cell occurred, as evidenced by the generation of a number of large neurospheres in the 33-week old HD but not the WT hippocampus.

Previously, it has been shown in the 20-week HD hippocampus, that there was a volume loss in the dentate gyrus as well as a significant decrease in both the number and percentage of NeuN/BrdU double-positive cells, however there was no significant decrease in the number of hippocampal-derived neurospheres [11]. In agreement with this, we also observed no significant difference in the number of neurospheres generated from the hippocampus of either 16- or 22-week-old HD and WT mice. Interestingly, at the much later time point of 33 weeks, although we observed a significant increase in the number of neurospheres generated from the HD hippocampus, we saw a significant decrease in BrdU-positive, DCX-positive and BrdU/DCX double-positive cells. Surprisingly, in a previous study, Lazic and colleagues observed maintenance of BrdU-positive cells at 25 weeks [12], [20], which differed from our findings at 33 weeks. If correct, this could indicate some activation of the precursors in HD animals around this stage, which would account for the increase in neurospheres we observed in the 30- and 33-week-old HD hippocampus.

One possible explanation for the increase in neurosphere numbers observed in the 33-week-old HD hippocampus is that the degenerating hippocampal environment present during HD progression may in fact lead to the release of factors *in vivo* which may help to maintain the stem/progenitor cell pool at its normal adult level, but not allow these cells to actively proliferate. This would explain why we observed no increase in neurogenesis in the HD hippocampus *in vivo*. If cells are primed by the degenerating environment present in HD progression but do not have the appropriate signals to proliferate there will be no increase in neurogenesis. It may be that once these primed precursor cells are cultured *in vitro* in the presence of the appropriate growth factors that they can then proliferate to form neurospheres. This could explain why in the symptomatic HD brain, although there was loss of neurogenesis *in vivo*, there was maintenance of the precursor cells capable of being activated.

Although it is not known how many types of latent stem cell populations are present in the adult hippocampus to date we have shown two. One, which is activated directly by nor-epinephrine [21] and one, which is activated, indirectly, by high potassium through the release of other soluble factors [13]. Both of these are dependent on FGF-2 for their proliferation and show extensive capacity for self-renewal. Although there remains the possibility that there are other latent populations that are regulated by other cytokines these cells will not be activated under the neurosphere growth conditions used in these experiments. The question of whether the stem cells we have identified are totally restricted to the neural lineage is difficult to answer unequivocally since it may depend on providing the appropriate milieu, suffice to say only cells of a neural lineage are produced under the differentiation conditions used in this study.

In addition, the capacity of neural progenitor cells to differentiate into region-specific neurons is a very important issue. Only those neural stem cells that can differentiate into region-specific neurons to replace damaged neuron types have a valuable application in regenerative medicine. We have preliminary data showing that ablation of the latent stem cell population in the adult hippocampus (genetic ablation of Nestin-positive stem cells), results in a significant decrease (approximately 30%) in doublecortin-positive neurons in the hippocampus four weeks post ablation (Walker et al., unpublished data). Therefore, importantly, indicating that the latent stem cell population is capable of giving rise to region-specific hippocampal neurons.

In conclusion, this study has revealed that the hippocampus contains a population of progenitors capable of responding to a changing environment. The up-regulation of *in vitro* hippocampal progenitor activity observed in response to an advancing disease state demonstrates a potential source of cells that could be manipulated to replace degenerating neurons. This inability of precursors to be activated to produce neurons may be one of the fundamental mechanisms underpinning the loss of neurogenesis in HD. Further experiments will be able to determine exactly what blocks the production of neurons from the latent precursors in HD. This may not only allow the restoration of neurogenesis, but may also allow this process to be harnessed to repair other areas of neuronal cell loss.
